# Hormonal and inflammatory signatures of different mood episodes in bipolar disorder: a large-scale clinical study

**DOI:** 10.1186/s12888-023-04846-1

**Published:** 2023-06-20

**Authors:** Nan Lyu, Qian Zhao, Bingbing Fu, Jinhong Li, Han Wang, Fan Yang, Sitong Liu, Juan Huang, Xinwei Zhang, Ling Zhang, Rena Li

**Affiliations:** 1 Beijing Key Laboratory of Mental Disorders, National Clinical Research Center for Mental Disorders & National Center for Mental Disorders, Beijing Anding Hospital, Capital Medical University, Beijing, 100088 China; 2 Advanced Innovation Center for Human Brain Protection, Capital Medical University, Beijing, 100069 China; 3 Center for Brain Disorders Research, Capital Medical University & Beijing Institute of Brain Disorders, Beijing, 100069 China; 4 Beijing SmindU Medical Science & Technology Co., Ltd, Beijing, 100020 China; 5 Beijing Key Laboratory of Mental Disorders, Beijing Anding Hospital, Capital Medical University, 5 Ankang Hutong Road, Beijing, 100088 Xicheng China; 6 The National Clinical Research Center for Mental Disorders, Beijing Anding Hospital, Capital Medical University, 5 Ankang Hutong Road, Beijing, 100088 Xicheng China

**Keywords:** Bipolar disorder, Mood episode, Hormone, Stress, Sex

## Abstract

**Background:**

Bipolar disorder (BD) is characterized by intensive mood fluctuations. While hormones imbalance plays important role in the mood swings, it is unknown whether peripheral hormones profiles could differentiate the manic and depressive mood episodes in BD. In this study, we investigated the changes of various hormones and inflammatory markers across distinct mood episodes of BD in a large clinical study to provide mood episode-specific peripheral biomarkers for BD.

**Methods:**

A total of 8332 BD patients (n = 2679 depressive episode; n = 5653 manic episode) were included. All patients were in acute state of mood episodes and need hospitalization. A panel of blood tests were performed for levels of sex hormones (serum levels of testosterone, estradiol, and progesterone), stress hormones (adrenocorticotropic hormone and cortisol), and an inflammation marker (C-reactive protein, CRP). A receiver operating characteristic (ROC) curve was used to analyze the discriminatory potential of the biomarkers for mood episodes.

**Results:**

In overall comparison between mood episodes, the BD patients expressed higher levels of testosterone, estradiol, progesterone, and CRP (*P* < 0.001) and lower adrenocorticotropic hormone (ACTH) level (*P* < 0.001) during manic episode. The episode-specific changes of testosterone, ACTH, and CRP levels remained between the two groups (*P* < 0.001) after correction for the confounding factors including age, sex, BMI, occupation, marital status, tobacco use, alcohol consumption, psychotic symptoms, and age at onset. Furthermore, we found a sex- and age-specific impact of combined biomarkers in mood episodes in male BD patients aged ≥ 45 years (AUC = 0.70, 95% CI, 0.634–0.747), not in females.

**Conclusions:**

While both hormone and inflammatory change is independently associated with mood episodes, we found that the combination of sex hormones, stress hormones and CRP could be more effective to differentiate the manic and depressive episode. The biological signatures of mood episodes in BD patients may be sex- and age-specific. Our findings not only provide mood episode-related biological markers, but also better support for targeted intervention in BD treatments.

**Supplementary Information:**

The online version contains supplementary material available at 10.1186/s12888-023-04846-1.

## Background

Bipolar disorder (BD) is a chronic recurrent disorder characterized by mood swings, involving episodes of mania, hypomania, and alternating depression [1]. Globally, over 1% of the population is affected by BD. The disorder results in significant functional impairment and high mortality, imposing a heavy social and economic burden on individuals, families, and countries at large [2]. However, the etiology of BD is complex and not fully understood. Several studies have linked BD with sex hormone fluctuations [3], stress [4] and inflammatory response [5]. Circulating blood-derived markers related to pathophysiologic processes of BD have been reported to differ among mood episodes [6], but none have been translated into clinical tests for traits or episodes of BD. Therefore, to enrich the existing evidence, more and broader potential biomarkers need to be explored.

The widespread effects of gonadal hormones in the brain and pathophysiology of affective disorders, have become increasingly evident in recent decades. Women with BD are more vulnerable to suffering from depression and an increased risk of affective dysregulation during periods of hormonal fluctuation [7]. Estradiol is involved in a wide range of brain functions, including neurodevelopment, neuroplasticity, and anti-inflammatory effects, which have implications for emotions [8]. Progestogens have significant, though indirect, effects by converting into neuroactive molecules, such as estrogens and testosterone [9]. Testosterone has been found to participate in the pathological process of BD and has anxiolytic and antidepressant effects in humans and animals, which are known to benefit negative emotions [10]. Presently, the relationship between gonadal hormones and mood episodes is unclear, and some studies have suggested that it may be related to mood episodes [11].

The activity of the hypothalamic-pituitary-adrenal (HPA) axis can be modulated by gonadal hormones [12]. Cortisol and ACTH are important biological indicators of a dysfunctional HPA axis and have been included as routine clinical test items to evaluate the status of the axis. High levels of cortisol would induce cell dysfunction or reorganization of dendrites in neurons in the long run, ultimately leading to significant neuroanatomical changes [13]. These, in turn, increase sensitization and vulnerability to mood disorders [14]. Increased release of cortisol and ACTH have also been observed in patients with mood disorder [15–17]. However, research on stress hormones during different mood episodes of BD is limited.

C-reactive protein (CRP) is a major acute-phase plasma protein in the inflammatory response induced by pro-inflammatory cytokines [18]. CRP levels in the peripheral blood are significantly higher in BD patients than in healthy controls [19] and vary across mood episodes [20]. CRP level increases in depressive episodes and to a greater degree in manic episodes [21, 22]. As the data are varied, with studies showing an increase, decrease, or no changes in CRP levels, these need to be tested in larger samples.

The above-mentioned sex hormones and stress/inflammation indicators have been studied in BD, but evidence for the changes of these indicators across distinct episodes was limited and sample size were also small. As we hypothesized that each episode might have different biomarkers, in this study, we investigated episode-related hormones and inflammatory markers in a large cohort of BD to provide clues toward investigating the pathophysiological processes associated with mood episodes.

## Materials and methods

### Study sample

The study cohort was selected from inpatients at Beijing Anding Hospital Capital Medical University between January 2013 to December 2019. The inclusion criteria were: (1) patients diagnosed with BD according to the International Statistical Classification of Diseases and Related Health Problems-10th revision (ICD-10) diagnostic criteria. The diagnosis was made by an experienced attending physician and determined by another senior physician. All the participants enrolled in this study were in the acute phase of BD. According to the type of episode, patients were divided into manic and depressive episode groups; (2) availability of reliable medical records, without any important information missing. The exclusion criteria were as follows: (1) those diagnosed with other mental disorders such as schizophrenia and major depressive disorder ;2) those diagnosed with a mixed or hypomanic episodes of BD; (2) those with severe physical diseases, such as autoimmune diseases, severe liver and kidney dysfunction;3) long-term hospitalized patients (>6 months); 4) those with acute infection and CRP > 10 mg/dL; 5) those having drug or alcohol dependence or abuse issues; 6) pregnant and lactating women; 7) those taking hormones including sex steroids or glucocorticoid medications.

### Study protocol

This was a retrospective study. Medical data from electronic health records were transformed into the Observational Medical Outcomes Partnership Common Data Model and developed the Beijing-Hebei-Tianjin Mental Health big data platform, which has been described in previous studies [23, 24]. Clinical and demographic data were extracted from the medical record available in the database. Peripheral blood was drawn from all subjects within 24–72 h in the morning after admission. Biochemical parameters, including estrogen, testosterone, progesterone, cortisol, ACTH, and CRP levels, were extracted from the big data platform. Neuroendocrine analytes (estradiol, testosterone, progesterone, prolactin, ACTH and cortisol) were measured using Atellica® Solution assays (Siemens Healthineers, Germany). CRP was estimated by Image 800 immunochemistry system (Beckman Coulter, United States). Normal-range values of each indicator was presented in Supplemental Table 6. In stratified analysis, 18 and 45 years of age were used for grouping. Under age of 18 years is a significant period of growth and physical development that includes changes in metabolic and hormonal fluctuations [25]. In addition, endocrine changes characteristic of the onset of the perimenopause begin at around age 45 [26]. Therefore, the BD patients were subgrouped into three age groups using 18 and 45 years of age. All data carrying patient identity information were de-labeled.

### Statistical methods

Statistical Product and Service Solutions version 26.0 (SPSS, Inc., Chicago, IL, USA) was used to perform statistical analyses. All continuous variables in this study do not conform to normal distribution and homogeneity of variance. They were presented as median and quartile ranges. Kruskal-Wallis H-test was used to compare differences in laboratory indicators between the two groups. All categorical variables were expressed as numbers and percentages (%) and compared by chi-square test. Two-tailed p-values were used for all statistical analyses, and *P* < 0.05 was considered statistically significant. Stepwise logistic regression analysis was used to measure the impact of variables and further to construct regression models. Confounding factors in logistic regression included age, sex, BMI, occupation, marital status, tobacco use, alcohol consumption, psychotic symptoms and age at onset. Receiver operating characteristic (ROC) curve analysis was used to assess the discriminatory effectiveness of continuous variables and logistic regression models by calculating the area under the ROC curve (AUC). Maximizing Youden’s index (J; where J = sensitivity + specificity − 1) was calculated to determine the optimal cut-offs between sensitivity and specificity.

## Results

### Study population and clinical features

For all BD patients, the mean age was 36.5 years and the age at onset was 26.0 years. The demographic and clinical characteristics of the patients in depressive episode group and manic episode group were summarized in Table 1. Compared with patients in the depressive episode group, those with manic episodes were younger (median 32 vs. 34 years, *P* < 0.001), male dominant (45.9% vs. 42.3%, *P* = 0.002), and had earlier age at onset (23 vs. 24 years, *P* = 0.018). The manic group showed higher rate of tobacco usage (22.7% vs. 19.9%, *P* = 0.021) and psychotic symptoms (55.4% vs. 40.0%, *P* < 0.001), and a lower rate of marriage (47.2% vs. 51.4%, P < 0.001) compared to the depressive episode group. Sex, age, BMI [27], tobacco use and alcohol consumption [28] play an important role in affective symptoms and have a significant correlation with hormone and inflammation levels [29, 30]. Occupation and marital status may affect emotion and hormones by chronic stress [31]; age at onset and psychotic symptoms are the important clinical characteristic of mood disorder and are associated with hormonal and inflammatory disturbance [32, 33]. So, we considered age, sex, BMI, occupation, marital status, tobacco use, alcohol consumption, psychotic symptoms, and age at onset as confounding factors.

**Table 1:** Demographic features and biochemical parameters

Variables		Depressive episode (n = 2679)	Manic episode (n = 5653)	Z/χ2	*P value*
**Demographic features**					
Age (years)		34(24, 51)	32(25, 45)	-5.193	**< 0.001**
Male (%)		1134/2679 (42.3)	2597/5653 (45.9)	9.585	**0.002**
Alcohol consumption (n/N, %)		127/1720 (7.4)	333/3735 (8.9)	3.384	0.066
Tobacco use (n/N, %)		343/1722 (19.9)	852/3746 (22.7)	5.515	**0.021**
Level of education (n, %)				1.949	0.583
	Elementary school or below	95/1722 (5.5)	191/3741 (5.1)		
	Secondary school	354/1722 (20.6)	803/3741 (21.5)		
	High school	814/1722 (47.3)	1710/3741 (45.7)		
	University or above	459/1722 (26.7)	1073/3741 (27.7)		
Occupation (n,%)				53.29	**< 0.001**
	Students	231/2679 (8.6)	385/5653(6.8)		
	Farmer/Worker	1970/2679 (73.5)	4274/5653(75.6)		
	Retirement	197/2679 (7.4)	242/5653(4.3)		
	Unemployed	281/2679 (10.5)	752/5653(13.3)		
Marital status (yes, n, %)		1377/2679 (51.4)	2659/5653(47.2)	13.68	**< 0.001**
BMI (kg/m^2^)		23.9 (21.2, 27.0)	24.0 (21.2, 27.2)	0.884	0.377
**Clinical Characteristics**					
Age at onset		24(17, 34)	23(18, 30)	-2.375	**0.018**
Duration of illness (years)		7(3, 15)	7(2, 14)	-1.861	0.063
Psychotic symptoms (n.%)		1071/2679(40.0)	3131/5653 (55.4)	172.02	**< 0.001**
Number of hospitalizations within 5 years(n.%)				3.240	0.198
	≤ 2 times	2243/2679 (83.7)	4816/5653 (85.2)		
	3–5 times	386/2679 (14.4)	748/5653 (13.2)		
	6–10 times	50/2679 (1.9)	89/5653 (1.6)		
**Biochemical Parameters**					
Testosterone (ng/dL)		58.8 (29.2, 326.3)	73.0 (35.0, 387.8)	6.824	**< 0.001**
Estradiol (pg/mL)		30.5 (18.6, 54.2)	36.03 (23.0, 59.9)	8.999	**< 0.001**
Progesterone (ng/mL)		0.49 (0.28, 0.85)	0.55 (0.32, 1.01)	7.104	**< 0.001**
Cortisol (µg/dL)		17.0 (13.2, 21.3)	17.1 (13.0, 21.4)	0.217	0.828
ACTH (pg/mL)		42.4 (28.3, 65.5)	39.3 (25, 61.8)	-5.016	**< 0.001**
CRP (mg/dL)		0.21 (0.16, 0.35)	0.26 (0.18, 0.45)	12.04	**< 0.001**
**Treatment strategies**					
Antipsychotics (n, %)		2186/2679 (81.6)	4629/5653 (81.9)	0.083	0.773
	Quetiapine	618 (28.3)	1243 (26.9)		
	Olanzapine	526 (24.1)	1100 (23.8)		
	Risperidone	404 (18.5)	855 (18.5)		
	Aripiprazole	244 (11.2)	547 (11.8)		
	Amisulpride	161 (7.4)	381 (8.2)		
	Clozapine	153 (7.0)	337 (7.2)		
	Paliperidone	125 (5.7)	273 (5.9)		
	Ziprasidone	18 (0.82)	47 (1.0)		
	Typical Antipsychotics	89 (4.1)	177 (3.8)		
Mood Stabilizers (n, %)		1171/2679 (43.7)	2465/5653 (43.6)	0.04	0.947
	Valproate	910 (77.7)	1892 (76.8)		
	Lithium	626 (53.5)	1350 (54.8)		
	Lamotrigine	15 (1.3)	53 (2.2)		
	Carbamazepine	1 (0.09)	4 (0.16)		
	Topiramate	1 (0.09)	1 (0.04)		
Antipsychotics combined with mood stabilizers (n, %)		1018/2258(47.4)	2274/4979(45.7)	0.216	0.642

Continuous data are presented as median (lower quartile, upper quartile) and compared by Kruskal–Wallis H test; Categorical data were indicated by (number/total number) and percentage (%) and compared by the Chi-square test

Abbreviations: CRP, C-reactive Protein; ACTH, Adrenocorticotropic Hormone; BMI, Body Mass Index

After correction by stepwise logistic regression, the impact of age, sex, and disease onset on the mood episodes still remained between the two groups, as shown in Table 2. For treatments, we found no difference in the usages of mood stabilizers, antipsychotics and combination of mood stabilizers and antipsychotics between the two mood episode groups (*P*>0.05) as shown in Table 1. A total of 830 BD patients during depressive episode used antidepressants, and the top five were sertraline (n = 233), escitalopram oxalate (n = 205), mirtazapine (n = 130), venlafaxine (n = 110), and duloxetine (n = 80).

**Table 2:** Stepwise logistic regression results between depressive and manic groups

Variables	B	Standard Error	Wald χ2	*P* value	OR	95%CI
Age	-0.014	0.003	22.33	**< 0.001**	0.986	0.980 ~ 0.992
Sex	-0.488	0.120	16.39	**< 0.001**	0.614	0.485 ~ 0.778
Age of onset	-0.01	0.004	5.909	**0.015**	0.990	0.982 ~ 0.998
Occupation*	—	—	22.22	**< 0.001**	—	—
Marital status (yes)	0.137	0.060	5.133	0.023	1.147	1.019 ~ 1.291
Tobacco use	-0.704	0.623	1.277	0.258	0.495	0.146 ~ 1.677
Alcohol consumption	0.132	0.124	1.134	0.287	1.141	0.895 ~ 1.456
Psychotic symptoms (yes)	-0.708	0.061	133.5	**< 0.001**	0.493	0.437 ~ 0.555
BMI (kg/m^2^)	0.010	0.006	2.824	0.093	1.010	0.998 ~ 1.023
Testosterone (ng/dL)	0.002	0.000	39.79	**< 0.001**	1.002	1.001 ~ 1.002
Estradiol (pg/mL)	0.000	0.000	0.009	0.924	1.000	0.999 ~ 1.001
Progesterone (ng/mL)	0.021	0.013	2.432	0.119	1.021	0.995 ~ 1.048
ACTH (pg/mL)	-0.003	0.001	12.89	**< 0.001**	0.997	0.996 ~ 0.999
CRP (mg/dL)	0.276	0.070	15.43	**< 0.001**	1.318	1.148 ~ 1.513

Abbreviations: SE, Standard Error; BMI, Body Mass Index; CRP, C-reactive Protein; ACTH, Adrenocorticotropic Hormone; OR: Odds Ratio; CI: Confidence Interval

^a^ Depression group as reference compared with mania group

Note: *Occupation is a multi-classification unordered variable and only the overall statistical results were listed in the table

### Mood episode-related biochemical parameters

To investigate the mood episode-related biochemical parameters, we evaluated and compared sex and stress hormone, and CRP levels in BD patients with depressive and manic episodes. As shown in Table 1, patients in manic episodes showed higher levels of testosterone, estradiol, progesterone, and CRP (*P* < 0.001) and lower levels of ACTH (*P* < 0.001) than those in depressive episodes. To further verify the relationship between biochemical changes and mood episodes, we performed logistic regression analysis and found that after controlling for confounding factors such as demographic and clinical features, the significant linkage between testosterone, ACTH, CRP, and mood episodes remained statistically significant (*P* < 0.001, Table 2).

### Age- and sex-specific biochemical characteristics of mood episodes

To explore the biochemical characteristics of mood episodes according to demographic and clinical features, stratified analysis was conducted. Features associated with mood episodes were screened (shown in Table 2). Comparisons of biochemical analytes stratified by selected parameters, including sex, age, age at onset, and presence of psychotic symptoms, were conducted. The results were presented in Supplemental Tables 1–4 respectively. Among the different features mentioned above, sex and biological age were seen to have effects on gonadal hormones, stress, and inflammatory markers. Therefore, refined stratification combined with age and sex was further carried out (Table 3).

**Table 3:** Results of comparisons of biochemical parameters stratified by age between depressive and manic episode

Groups	n	Testosterone (ng/dL)	Estradiol (pg/mL)	Progesterone (ng/mL)	Cortisol (µg/dL)	ACTH (pg/mL)	CRP (mg/dL)
***Male***							
** <18 years**							
Depression	79	436.6 (322.5, 537.8)	28.2 (20.1, 37.4)	0.44 (0.29, 0.72)	16.8 (13.2, 19.7)	52.5 (38.3, 74.0)	0.20 (0.15, 0.26)
Mania	164	392.9 (306.7, 551.3)	28.6 (20.6, 36.2)	0.49 (0.35, 0.77)	17.7 (13.2, 21.6)	57.5 (32.0, 77.9)	0.22 (0.15, 0.34)
Z		-0.734	-0.144	1.174	1.202	0.183	-0.217
*P* value		0.463	0.885	0.240	0.229	0.855	0.828
**18 ~ 44 years**							
Depression	737	377.1 (269.9, 521.2)	27.5 (19.2, 36.7)	0.51 (0.33, 0.79)	16.6 (12.7, 20.6)	48.8 (31.0, 71.8)	0.21 (0.16, 0.36)
Mania	1814	396.8 (286.0, 530.1)	31.8 (23.1, 42.4)	0.58 (0.36, 0.91)	17.5 (13.6, 21.6)	46.9 (30.0, 68.0)	0.27 (0.19, 0.47)
*Z*		2.523	7.537	4.017	3.365	-1.196	7.391
*P* value		**0.012**	**< 0.001**	**< 0.001**	**0.001**	0.232	**< 0.001**
**≥ 45 years**							
Depression	318	341.1 (248.4, 476.7)	24.3 (17.5,32.9)	0.39 (0.23, 0.56)	17.6 (14.3, 20.7)	49.6 (34.0, 73.5)	0.21 (0.16, 0.34)
Mania	619	439.0 (313.0, 589.3)	30.9 (21.8, 41.1)	0.43 (0.27, 0.63)	16.2 (12.8, 20.8)	41.8 (27.2, 64.4)	0.31 (0.21, 0.60)
Z		6.880	6.380	2.456	-2.784	-4.520	7.716
*P* value		**< 0.001**	**< 0.001**	**0.014**	**0.005**	**< 0.001**	**< 0.001**
***Female***							
**<18 years**							
Depression	103	31.8 (21.7, 50.4)	56.5 (32.3, 116.9)	0.56 (0.33,1.05)	16.0 (12.3, 21.6)	41.4 (27.9, 65.8)	0.17 (0.14, 0.24)
Mania	123	37.9 (23.5, 51.5)	54.8 (32.1, 95.2)	0.58 (0.39,1.14)	16.7 (12.7, 20.9)	34.5 (20.3, 54.5)	0.20 (0.15,0.30)
Z		1.414	-0.594	0.412	0.374	-1.743	2.536
*P* value		0.157	0.552	0.681	0.709	0.081	**0.011**
**18 ~ 44 years**							
Depression	865	37.7 (25.6, 52.9)	61.3 (33.9,106.4)	0.76 (0.37, 2.30)	16.5 (12.3, 21.3)	35.8 (23.6, 55.4)	0.20 (0.14, 0.32)
Mania	2072	41.2 (28.4, 56.9)	61.4 (36.7,114.4)	0.80 (0.40, 2.45)	17.3 (12.9, 21.6)	34.8 (23.0, 56.8)	0.23 (0.16,0.39)
*Z*		4.292	1.764	1.425	1.586	-0.356	5.563
*P* value		**< 0.001**	0.078	0.154	0.113	0.722	**< 0.001**
**≥ 45 years**							
Depression	577	25.1 (20.0, 37.8)	15.4 (5.00, 31.2)	0.31 (0.20,0.52)	18.0 (13.8, 22.3)	41.7 (28.0, 63.3)	0.24 (0.17, 0.39)
Mania	861	25.9 (20.0, 38.7)	17.9 (5.73, 39.1)	0.34 (0.20,0.58)	16.5 (12.1, 20.8)	31.1 (20.3, 48.5)	0.29 (0.20, 0.52)
Z		0.949	2.275	2.174	-4.694	-7.382	5.083
*P* value		0.343	**0.023**	**0.030**	**< 0.001**	**< 0.001**	**< 0.001**

Abbreviations: CRP, C-reactive Protein; ACTH, Adrenocorticotropic Hormone

For patients younger than 18 years of age, there were no significant differences in gonadal hormones, stress hormones, and CRP between depressive and manic episodes, regardless of sex (Tables 3 and 4). In women younger than 18 years, CRP levels (Z = 2.536, *P* = 0.011) were higher in manic episodes than in depressive episodes; however, this difference was not observed in the logistic regression model (Table 4).

**Table 4:** Stepwise logistic regression results between depressive and manic episodes stratified by age and sex

*Male*	< 18 years (n = 243)^a^						18 ~ 44 years (n = 2551)^a^							≥ 45 years (n = 937)^a^					
Variables	B	Waldχ2	*P*	OR	95%CI		B		Waldχ2	*P*	OR	95%CI		B	Wald χ2	*P*	OR	95%CI	
Testosterone	—	—	0.680	—	—		—		—	0.389	—	—		0.002	26.85	**< 0.001**	1.002	1.002 ~ 1.003	
Estradiol	—	—	0.848	—	—		0.025		57.86	**< 0.001**	1.026	1.019 ~ 1.032		0.017	7.891	**0.005**	1.018	1.005 ~ 1.029	
Progesterone	—	—	0.238	—	—		0.423		12.83	**0.001**	1.604	1.251 ~ 2.149		0.665	6.165	**0.012**	1.965	1.161 ~ 3.326	
Cortisol	—	—	0.202	—	—		0.026		4.838	**0.029**	1.022	1.008 ~ 1.042		—	—	0.202	—	—	
ACTH	—	—	0.483	—	—		-0.003		59.10	**0.004**	0.997	0.995 ~ 0.999		-0.006	8.447	**0.004**	0.994	0.990 ~ 0.998	
CRP	—	—	0.349	—	—		0.209		3.943	**0.047**	1.232	1.003 ~ 1.513		0.573	11.462	**0.001**	1.773	1.273 ~ 2.470	
***Female***	**< 18 years (n = 226)** ^**a**^						**18 ~ 44 years (n = 2937)** ^**a**^							**≥ 45 years (n = 1439)** ^**a**^					
Variables	B	Waldχ2	*P*	OR	95%CI		B	Waldχ2		*P*	OR	95%CI		B	Waldχ2	*P*	OR	95%CI	
Testosterone	—	—	0.162	—	—		0.008	17.20		**< 0.001**	1.008	1.004 ~ 1.012		—	—	0.270	—	—	
Estradiol	—	—	0.103	—	—		—	—		0.643	—	—		—	—	0.525	—	—	
Progesterone	—	—	0.358	—	—		—	—		0.509	—	—		0.140	9.333	**0.002**	1.150	1.051 ~ 1.257	
Cortisol	—	—	0.526	—	—		—	—		0.720	—	—		-0.019	4.803	**0.028**	0.981	0.964 ~ 0.998	
ACTH	—	—	0.296	—	—		—	—		0.722	—	—		-0.009	21.43	**< 0.001**	0.990	0.988 ~ 0.995	
CRP	—	—	0.477	—	—		—	—		0.092	—	—		—	—	0.056	—	—	

Abbreviations: CRP, C-reactive Protein; ACTH, Adrenocorticotropic Hormone; OR: Odds Ratio; CI: Confidence Interval

^a^ Depression group as reference compared with mania group

For male patients aged 18–44 years, indicators during manic and depressive episodes differed in terms of testosterone (Z = 2.523, *P* = 0.012), estradiol (Z = 7.537, *P* < 0.001), progesterone (Z = 4.017, *P* < 0.001), cortisol (Z = 3.365, *P* = 0.001) and CRP (Z = 7.391, *P* < 0.001). Progesterone, estradiol, cortisol, and CRP levels were remained in the stepwise logistic regression model (Table 4). In female patients aged 18–44 years, testosterone (Z = 4.292, *P* < 0.001) and CRP (Z = 5.563, *P* < 0.001) were higher in manic episodes than in depressive episodes. Logistic regression analysis revealed that testosterone levels still had an effect in mood episodes. Female hormones were not associated with mood episodes.

In male patients aged 45 years or above, sex hormones (testosterone, estradiol, and progesterone), stress hormones (cortisol and ACTH), and CRP levels were different between the mood episodes (*P* < 0.005), as depicted in Table 3. The differences in testosterone, estradiol, progesterone, ACTH, and CRP levels were still noted after stepwise logistic regression analysis (Table 4). In female patients aged 45 years or above, higher levels of estradiol (Z = 2.275, *P* = 0.023), progesterone (Z = 2.174, *P* = 0.03), and CRP (Z = 5.083, *P* < 0.001), as well as lower levels of cortisol (Z=-4.694, *P* < 0.001) and ACTH (Z=-7.382, *P* < 0.001), were found during manic episodes in comparison with depressive episodes (Table 3). Progesterone, cortisol, and ACTH were further included in the logistic regression model (Table 4).

### Potential discriminatory markers of mood episodes

To discriminate each episode, ROC analysis was conducted to investigate the efficacy of characteristic variables acting as mood-specific biomarkers. For the different age and sex subgroups, ROC analysis was performed separately, and the results were shown in Supplemental Table 5. Each single indicator of the subgroups was not specific enough to differentiate mood episodes, with an AUC ranging from 0.506 to 0.654. The combination model of several indicators in the subgroups improved discriminatory effectiveness, with an AUC value ranging from 0.500 to 0.700. Interestingly, for male patients aged 45 years or above, the combined model of five indicators (testosterone, estradiol, progesterone, ACTH, and CRP) could discriminate mood episodes with an AUC of 0.700 (*P* < 0.001) (Table 5).

**Table 5:** Results of ROC analysis between depressive and manic groups in males over the age of 45 years

Variables	AUC	Cutoff	SE	*P* value	95% CI^a^	Sensitivity	Specificity	LR+	LR-
**Males aged ≥ 45 years**									
Testosterone (ng/dL)	0.628	393.2	0.037	0.001	0.556 ~ 0.700	65.56	62.50	1.748	0.551
Estradiol (pg/mL)	0.637	32.21	0.037	< 0.001	0.566 ~ 0.709	73.33	51.39	1.509	0.519
Progesterone(ng/mL)	0.549	0.215	0.020	0.0141	0.549 ~ 0.600	78.90	30.00	1.126	0.705
ACTH (pg/mL)	0.579	48.20	0.022	< 0.001	0.496 ~ 0.644	61.11	53.33	1.309	0.729
CRP (mg/dL)	0.654	0.235	0.018	< 0.001	0.606 ~ 0.702	62.58	62.84	1.684	0.596
Combined model	**0.700**	0.632	0.018	< 0.001	0.634 ~ 0.747	64.78	67.37	1.985	0.523

Note: ^a^ 95% CI for AUC. Abbreviations: AUC, Area Under the Curve; LR+, Positive Likelihood Ratio; LR-, Negative Likelihood Ratio

## Discussion

We included 8 332 BD patients and compared their sex hormones, stress, and inflammatory indicators to explore the biochemical signatures across different mood episodes. The results revealed that, in contrast to depressive episodes, manic episodes were associated with higher levels of testosterone and CRP, and lower levels of ACTH. Interestingly, we also noticed a sex- and age-difference in the peripheral biomarkers, the impact of combined biomarker-associated mood episodes was only found in male BD patients aged 45 years or above.

In our study cohort, the mean age of all BD patients was 36.5 years and the age at onset was 26.0 years, which conforms to recent epidemiological findings that the prevalence of BD was higher in patients aged 35–49 years (0.6%) [34] and the age of onset ranged from 21 to 33 years [35, 36]. There were more female than male patients in our study (55.2% vs. 44.8%), which is supported by some studies that found an increased prevalence of BD in women [37, 38], although some studies reported no sex difference in BD prevalence [34, 39]. In addition, our cohort included more patients with manic episodes, in which the proportion of male patients was higher than that of depressive episodes. This may be due to the bias from inclusion of hospitalized patients. Manic patients, especially males, are more difficult to take care of by their relatives because of their impulsivity, aggressiveness, and impaired judgment [40]. Therefore, more patients with manic episodes are admitted to the hospital, whereas patients with depressive episodes are likely to choose outpatient treatment.

The depressive and manic phases appear to be associated with different gonadal hormone profiles. Previous studies have found that testosterone levels are higher during manic episodes and lower during depressive episodes in contrast to controls [41–44]. The findings of our study are similar. Higher baseline testosterone levels were also reported to correlate with aggressive behavior in patients and may act as a predictor of suicide attempts during follow-up [45, 46]. In addition, the levels of testosterone reflect the effect on modulating motivation and competitive behaviors [47]. This may partly explain why testosterone levels are higher in manic patients with elevated, expansive, or irritable mood. In our study, estradiol and progesterone levels differed between depressive and manic episodes when an overall comparison was drawn, but the differences were not visible after controlling for the confounding factors. This suggests that estradiol and progesterone are probably affected by other factors such as age and sex.

Our results indicate that mood episodes related gonadal hormones are sex- and age-specific. In patients younger than 18 years of age, gonadal hormones had no relationship with mood episodes regardless of sex. Adolescence is a unique plasticity window strongly influenced by gonadal hormones, but no effects of these hormones on mood episodes were observed in our study. One possibility is that there are hormonal changes in both depressive and manic episode groups, and the difference is not significant between the two groups. Another reason is the relatively small sample size. In our study, for male patients aged 18–44 years, all sex hormones were associated with mood episodes, while in females, only testosterone showed a relationship with mood episodes. Testosterone may act as a mood episode-specific biomarker in adults regardless of sex. For middle-aged and elderly patients, testosterone, estradiol, and progesterone levels differed between depressive and manic episodes in men, whereas only progesterone levels were different in women. Studies have shown that increased estradiol levels are associated with depression in elderly men [48], although adult men show considerably lower total serum estradiol levels with minimal fluctuations and age-related decline [49]. Women aged 45 years or above are likely to experience menopause with hormonal fluctuations, which decreases mood stability [50]resulting in mood episodes.

Stress and inflammatory activity also have an influence on bipolar patients with symptomatic episodes of BD. Elevated cortisol is among the most robust pathophysiological findings in mood disorders [14, 51].Increased ACTH levels have been documented to predict ongoing depressive symptoms and the severity of depression [52] and is also reported to be one of the most predictive biomarkers of suicide [53]. This evidence suggests that ACTH and cortisol may have an impact on mood episodes, and this association was further confirmed in our study. In addition, this correlation is particularly evident in elderly women. In female patients aged 45 years or above, higher ACTH and cortisol levels were observed in the depressive phase than in the manic phase. However, this phenomenon was not observed in young women (< 18 years and 18–45 years). Previous studies have demonstrated that life stress is strongly associated with perimenopause [54]. The maintenance of high levels of cortisol may exert neurotoxic effects and affect emotion [55]. Some evidence supports the association between the depressive phase and heightened cortisol levels, with the absence of cortisol dysfunction during mania and euthymic periods of remission [14].

Differences in inflammatory cytokines across mood episodes have been observed [56–58]. In our study, we found that CRP were higher in manic phase than depressive phase. Previous studies support our results. A meta-analysis including 27 studies with 84,093 participants showed that CRP levels were moderately elevated in BD patients during depression and euthymia, and more significantly increased during mania [20].

We found that in men aged 45 years or above, gonadal hormones (testosterone, estradiol, progesterone), and stress/inflammatory indicators (ACTH, cortisol, and CRP) were moderately affected (AUC = 0.70) in distinguishing mood episodes. The characteristics of mood episodes in BD patients may be sex- and age-specific. Previous study also found that CRP in BD patients may sex-dependent [59].In men aged 45 years or above, gonadal hormones and stress indicators had a greater influence on mood episodes. The interaction between the hypothalamic-pituitary-gonadal (HPG) and HPA axes may be involved in mood episodes by regulating stress and a range of emotional and social behaviors [60]. The combination of these axes as indicators may be a promising approach to assist differentiating mood episodes according to sex and age.

In addition to the signatures of hormone and inflammatory at the manic and depressive episode, we are also going to follow up these patients and further identify pathways or models to predict the onset of manic or depressive episodes in the future, which will be of great benefit to patients and early appropriate intervention can be conducted.

Indeed, there are different underlying mechanisms and pathways in the distinct episodes of BD. Early pilot studies have discovered genes involved in myelination and growth factor signaling which might be related to mood states [61, 62], such as the gene expression of *FGFR1*, *MAG*, *PMP22*, *UGT8* AND *ERBB3* for low mood and *MBP* and *EDNRB* for high mood. Subsequent genes related to circadian, neurotrophic, cell differentiation, serotonergic and glutamatergic signaling in mood episodes were also reported [63], such as *NRG1*, *DOCK10*, *GLS*, *PRPS1*, *TMEM161B*, *GLO1*, *FANCF*, *HNRNPDL*, *CD47*, *OLFM1*, *SMAD7*, and *SLC6A4* for depression, while *RLP3* and *SLC6A4* for mania. In addition, an opposite change of neurotrophies levels were also found in manic and depressive episodes of BD patients, such as increased levels of neurotrophin-3 and neurotrophin-4 during depressive episode, while decreased levels during the manic episode [64]. Furthermore, Pro and anti-inflammatory cytokine showed different profiles during distinct episodes of BD. For example, the IL-2 levels elevated in manic episode and reduced in depressive episode in some studies while other report showed a decreased IL-2 in the manic episode [21, 65, 66]. Other cytokines as IL-4 and IL-6 are also involved in the mood episodes although the relationship between these cytokines and mood episodes are controversial [21, 65–69]. Together, it is important to explore potential mechanisms of mood episodes using multiple biomarkers from different perspectives in the future.

This study has several notable strengths. First, it comprised a relatively large sample of BD patients with fine subgroups according to sex and age. Thus, indicators related to mood episodes in different subpopulations could be better explored. In addition, the diagnosis of inpatients was confirmed by at least two senior doctors during ward rounds and long-term observation. Finally, all blood samples were processed in the same manner in the same laboratory, thus minimizing the impact of measurement deviations. Nevertheless, our study also has several limitations. First, a healthy control group could not be established, as the hospital medical database did not include a healthy population. But the parameters involved in the study have been shown to differ between BD patients and healthy controls in previous studies; therefore, we further characterized the signature of neuroendocrine biochemical indicators across different mood episodes of BD. Second, the potential effects of psychiatric medications were not considered in our study. Third, the severity of symptoms with detailed assessments of scales were lacking. Therefore, we were unable to establish a relationship between scale scores and hormone levels. Additionally, longitudinal studies should be conducted to explore whether mood episode-related biomarkers can predict the pattern of mood episodes in the future.

## Conclusions

In conclusion, our findings demonstrated significant differences in the levels of sex hormones, stress, and inflammation between the manic and depressive phases. Combination of sex hormones, stress hormones and CRP could be more effective to differentiate the sex- and age-specific manic and depressive episode. Our findings not only provide mood episode-related biological markers, but also better support for targeted intervention in bipolar disorder treatments.

## Electronic supplementary material

Below is the link to the electronic supplementary material.

Additional file 1

## Data Availability

The datasets generated and analyzed during the current study are not publicly available due to ethical restrictions and personal data protection, but are available from the corresponding authors Rena Li and Ling Zhang on reasonable request.

## Abbreviations

BD: Bipolar disorder
CRP: C-reactive protein
ACTH: Adrenocorticotropic hormone
ROC: A receiver operating characteristic curve
BDNF: Brain derived neurotrophic factor
HPA: Hypothalamic-pituitary-adrenal axis
HPG: Hypothalamic-pituitary-gonadal axis
ICD-10: International Statistical Classification of Diseases and Related Health Problems-10th revision
