# Supplementary material for: Hormonal and inflammatory signatures of different mood episodes in bipolar disorder: a large-scale clinical study
Source: BMC Psychiatry. 2023 Jun 20;23:449. doi: 10.1186/s12888-023-04846-1 (PMC10283309; doi:10.1186/s12888-023-04846-1)
Supplement: Supplementary file 1 — Additional file 1 [file 12888_2023_4846_MOESM1_ESM.docx]

Supplemental Materials

**Supplemental Tables**

**Supplemental Table 1.** **Results of comparisons of biochemical parameters stratified by sex.**

| Groups | n | Testosterone  (ng/ dL) | Estradiol  (pg/mL) | Progesterone  (ng/mL) | Cortisol  (μg/dL) | ACTH  (pg/mL) | CRP (mg/dL) |
| --- | --- | --- | --- | --- | --- | --- | --- |
| **Male** |  |  |  |  |  |  |  |
| depression | 1134 | 368.7 (264.4, 504.3) | 26.7 (19.1, 35.8) | 0.47 (0.30, 0.74) | 16.9 (13.3, 20.6) | 49.0 (32.6, 72.2) | 0.21 (0.16, 0.35) |
| Mania | 2597 | 407.7 (293.0, 543.7) | 31.4 (22.7, 41.6) | 0.53 (0.33, 0.84) | 17.2 (13.4, 21.4) | 46.2 (29.7, 67.8) | 0.28 (0.19, 0.49) |
| Z |  | 5.382 | 9.629 | 5.256 | 1.82 | -3.229 | 10.21 |
| *P* value |  | **<0.001** | **<0.001** | **<0.001** | 0.069 | **0.001** | **<0.001** |
| **Female** |  |  |  |  |  |  |  |
| depression | 1545 | 32.6 (20.7, 47.35) | 40.0 (17.5, 86.1) | 0.51 (0.26, 1.07) | 17.2 (13.0, 21.8) | 38.4 (25.5, 58.7) | 0.21 (0.15, 0.34) |
| Mania | 3056 | 37.0 (23.9, 52.7) | 49.4(24.0, 97.5) | 0.59 (0.31, 1.44) | 17.0 (12.6, 21.4) | 33.9(22.3, 54.3) | 0.24 (0.17, 0.42) |
| Z |  | 6.305 | 5.776 | 5.355 | -1.335 | -4.954 | 6.852 |
| *P* value |  | **<0.001** | **<0.001** | **<0.001** | 0.182 | **<0.001** | **<0.001** |

Abbreviations: CRP, C-reactive protein; ACTH, adrenocorticotropic hormone.

**Supplemental Table 2. Results of comparisons of biochemical parameters stratified by age**

| Groups | n | Testosterone  (ng/dL) | Estradiol  (pg/mL) | Progesterone  (ng/mL) | Cortisol  (μg/dL) | ACTH  (pg/mL) | CRP (mg/dL) |
| --- | --- | --- | --- | --- | --- | --- | --- |
| **＜18 years** |  |  |  |  |  |  |  |
| depression | 182 | 60.5 (29.0, 412.7) | 36.8 (24.7, 62.9) | 0.52 (0.31, 0.82) | 16.3 (12.5, 20,7) | 46.2(31.0, 68.3) | 0.18 (0.14, 0.25 ) |
| Mania | 287 | 225.6 (41.1, 430.0) | 33.5 (23.5, 53.5) | 0.53 (0.35, 0.87) | 17.3 (12.7, 21.3) | 43.3(27.5, 70.9) | 0.21 (0.15, 0.33 ) |
| Z |  | 2.565 | -1.575 | 0.687 | 1.132 | -0.517 | 2.566 |
| *P* value |  | **0.01** | 0.115 | 0.492 | 0.258 | 0.605 | **0.01** |
| **18~44years** |  |  |  |  |  |  |  |
| depression | 1602 | 73.7 (35.7, 350.3) | 37.1 (23.0, 65.8) | 0.61 (0.35, 1.08) | 16.5 (12.6, 21.0) | 40.6 (27.0, 63.7) | 0.21 (0.15,0.34 ) |
| Mania | 3886 | 81.4 (39.9, 381.1) | 41.1 (27.4, 69.5) | 0.66 (0.38, 1.19) | 17.3 (13.3, 21.6) | 40.8 (25.6, 63.1) | 0.25 (0.17,0.42 ) |
| *Z* |  | 2.866 | 4.927 | 3.327 | 3.453 | -0.751 | 9.052 |
| *P value* |  | **0.004** | **<0.001** | **0.001** | **0.001** | 0.453 | **<0.001** |
| **≥45 years** |  |  |  |  |  |  |  |
| depression | 895 | 40.0 (20.1, 262.4) | 20.0 (8.48, 32.4) | 0.33 (0.20,0.54 ) | 17.8 (13.9,21.9) | 44.7 (30.5,66.7) | 0.23 (0.17, 0.38) |
| Mania | 1480 | 47.2 (23.3, 401.8) | 24.7 (12.2, 40.5) | 0.38 (0.21,0.61) | 16.3 (12.5,20.8) | 35.6 (23.2,55.2) | 0.30 (0.20, 0.55) |
| Z |  | 4.666 | 5.663 | 3.677 | -5.409 | -7.696 | 8.829 |
| *P* value |  | **<0.001** | **<0.001** | **<0.001** | **<0.001** | **<0.001** | **<0.001** |

Abbreviations: CRP, C-reactive protein; ACTH, adrenocorticotropic hormone.

**Supplemental Table 3. Results of comparisons of biochemical parameters stratified by age of onset**

| Groups | n | Testosterone  (ng/dL) | Estradiol  (pg/mL) | Progesterone  (ng/mL) | Cortisol  (μg/dL) | ACTH  (pg/mL) | CRP (mg/dL) |
| --- | --- | --- | --- | --- | --- | --- | --- |
| **＜18 years** |  |  |  |  |  |  |  |
| depression | 599 | 65.4 (34.3,352.0 ) | 37.7(24.1,65.8 ) | 0.58 (0.34,0.97 ) | 16.8 (12.5,21.2 ) | 41.9 (28.4,65.5 ) | 0.20 (0.15,0.31 ) |
| Mania | 1106 | 201.6 (45.0,438.7 ) | 37.1 (25.1,59.3 ) | 0.64 (0.38,1.06 ) | 16.9(12.8,21.4) | 43.4(27.5,66.6 ) | 0.24(0.17,0.39 ) |
| Z |  | 5.331 | -0.252 | 1.859 | 0.889 | 0.225 | 5.344 |
| *P* value |  | **<0.001** | 0.801 | 0.063 | 0.374 | 0.822 | **<0.001** |
| **19~25 years** |  |  |  |  |  |  |  |
| depression | 557 | 107 (38.45,381.6 ) | 31.1 (19.6, 52.4 ) | 0.57 (0.32,0.97 ) | 17.2(13.2, 21.6) | 41.5(26.6,64.3 ) | 0.22 (0.16,0.37 ) |
| Mania | 1589 | 99.6 (39.8,391.3 ) | 39.0 (25.8, 63.1 ) | 0.64 (0.37, 1.13 ) | 17.4(13.3,21.8) | 38.7 (24.4,59.6 ) | 0.26 (0.18,0.45 ) |
| Z |  | 0.512 | 6.109 | 2.870 | 0.360 | -2.133 | 4.462 |
| *P* value |  | 0.608 | **<0.001** | **0.004** | 0.719 | **0.033** | **<0.001** |
| **26~49 years** |  |  |  |  |  |  |  |
| depression | 871 | 51.5 (26.5,300.0 ) | 29.1 (15.8,51.8 ) | 0.48 (0.28,0.83 ) | 17.5 (13.6,21.7 ) | 43.2 (29.7,65.8 ) | 0.21 (0.16,0.35 ) |
| Mania | 1733 | 53.2 (29.2,336.4 ) | 35.5(20.8,66.3 ) | 0.54 (0.32,0.98 ) | 17.3 (13.0,21.7 ) | 38.5 (24.4,59.1 ) | 0.27 (0.19,0.5 ) |
| Z |  | 1.858 | 5.866 | 4.380 | -0.947 | -4.549 | 8.512 |
| *P* value |  | 0.063 | **<0.001** | **<0.001** | 0.344 | **<0.001** | **<0.001** |
| **≥50 years** |  |  |  |  |  |  |  |
| depression | 164 | 40 (20.0, 196.1 ) | 16.5 (5.0, 26.0 ) | 0.31 (0.2,0.52 ) | 18.7 (14.1, 22.8 ) | 46.8 (30.6,75.8) | 0.24 (0.16,0.43 ) |
| Mania | 194 | 53.8 (22.5,447.0 ) | 20.8 (9.89,33.8 ) | 0.37 (0.21,0.58 ) | 16.6(12.7,21.3) | 33.4 (22.6,55.7 ) | 0.34 (0.21,0.63 ) |
| Z |  | 2.929 | 2.885 | 1.892 | -2.417 | -4.415 | 3.891 |
| *P* value |  | **0.003** | **0.004** | 0.059 | **0.016** | **<0.001** | **<0.001** |

Abbreviations: CRP, C-reactive protein; ACTH, adrenocorticotropic hormone.

**Supplemental Table 4. Results of comparisons of biochemical parameters stratified by psychotic symptoms.**

| Groups | n | Testosterone  (ng/dL) | Estradiol  (pg/mL) | Progesterone  (ng/mL) | Prolactin  (ng/mL) | Cortisol  (μg/dL) | ACTH  (pg/mL) | CRP (mg/dL) |
| --- | --- | --- | --- | --- | --- | --- | --- | --- |
| **Without psychotic symptoms** | | |  |  |  |  |  |  |
| depression | 1608 | 57.6 (28.6, 344.2) | 30.0 (17.5,51.5) | 0.46 (0.26,0.79) | 21.1 (12.7, 36.6) | 16.7 (12.6, 20.7) | 42.9 (29.0, 65.5) | 0.21 (0.15, 0.34) |
| Mania | 2522 | 108.7 (35.4, 419.9) | 35.6 (23.1,57.2) | 0.52 (0.31,0.96) | 36.1 (21.3, 64.3) | 16.5(12.6, 20.9) | 39.7 (24.8, 61.1) | 0.25 (0.17, 0.43) |
| Z |  | 6.512 | 7.077 | 5.349 | 17.91 | -0.399 | -4.614 | 8.955 |
| *P* value |  | **<0.001** | **<0.001** | **<0.001** | **<0.001** | 0.690 | **<0.001** | **<0.001** |
| **With psychotic symptoms** | | |  |  |  |  |  |  |
| depression | 1071 | 61.0 (30.2, 306.0) | 31.7 (19.7, 57.6) | 0.52 (0.30, 0.90) | 28.6 (14.3, 55.0) | 17.5 (13.8, 22.2) | 41.4 (27.2, 65.5) | 0.22 (0.16, 0.36) |
| Mania | 3131 | 65.9 (34.7, 355.1) | 36.4 (23.0,62.6) | 0.58 (0.33, 1.06) | 41.5 (24.7, 71.9) | 17.6 (13.4, 21.8) | 39.2 (25.1, 62.5) | 0.26 (0.18, 0.46) |
| Z |  | 3.437 | 4.928 | 3.465 | 10.916 | -0.907 | -2.207 | 7.210 |
| *P* value |  | **0.001** | **<0.001** | **0.001** | **<0.001** | 0.365 | **0.027** | **<0.001** |

Abbreviations: CRP, C-reactive protein; ACTH, adrenocorticotropic hormone.

**Supplemental Table 5. Results of ROC analysis stratified by sex and age.**

| Variables | AUC | SE | *P* value | 95% CI |
| --- | --- | --- | --- | --- |
| **Male ＜18 years** |  |  |  |  |
| Testosterone (ng/dl) | 0.529 | 0.039 | 0.463 | 0.453~0.605 |
| Estradiol (pg/mL) | 0.506 | 0.039 | 0.885 | 0.430~0.582 |
| Progesterone (ng/mL) | 0.547 | 0.040 | 0.240 | 0.467~0.626 |
| Cortisol (μg/dL) | 0.548 | 0.038 | 0.229 | 0.473~0.622 |
| ACTH (pg/mL) | 0.507 | 0.039 | 0.855 | 0.431~0.583 |
| CRP(mg/dL) | 0.535 | 0.039 | 0.376 | 0.459~0.611 |
| Combined model | 0.500 | 0.040 | 1.000 | 0.522~0.675 |
| **Male 18~45 years** |  |  |  |  |
| Testosterone (ng/dl) | 0.532 | 0.013 | 0.012 | 0.507~0.557 |
| Estradiol (pg/mL) | 0.595 | 0.012 | <0.001 | 0.571~0.619 |
| Progesterone (ng/mL) | 0.551 | 0.012 | <0.001 | 0.527~0.575 |
| Cortisol (μg/dL) | 0.542 | 0.013 | 0.001 | 0.518~0.567 |
| ACTH (pg/mL) | 0.485 | 0.013 | 0.232 | 0.460~0.510 |
| CRP(mg/dL) | 0.593 | 0.013 | <0.001 | 0.569~0.618 |
| Combined model | 0.625 | 0.012 | <0.001 | 0.602~0.649 |
| **Male ≥ 45 years** |  |  |  |  |
| Testosterone (ng/dl) | 0.628 | 393.2 | 0.001 | 0.556~0.700 |
| Estradiol (pg/mL) | 0.637 | 32.21 | <0.001 | 0.566~0.709 |
| Progesterone (ng/mL) | 0.549 | 0.215 | 0.0141 | 0.549~0.600 |
| Cortisol (μg/dL) | 0.556 | 0.019 | 0.005 | 0.506-0.605 |
| ACTH (pg/mL) | 0.579 | 48.2 | <0.001 | 0.496~0.644 |
| CRP(mg/dL) | 0.654 | 0.235 | <0.001 | 0.606~0.702 |
| Combined model | 0.700 | 0.632 | <0.001 | 0.634~0.747 |
| **Female ＜18 years** |  |  |  |  |
| Testosterone (ng/dl) | 0.555 | 0.039 | 0.157 | 0.479~0.63 |
| Estradiol (pg/mL) | 0.523 | 0.039 | 0.552 | 0.446~0.599 |
| Progesterone (ng/mL) | 0.516 | 0.039 | 0.681 | 0.44~0.592 |
| Cortisol (μg/dL) | 0.514 | 0.039 | 0.709 | 0.438~0.591 |
| ACTH (pg/mL) | 0.576 | 0.038 | 0.081 | 0.492~0.643 |
| CRP(mg/dL) | 0.598 | 0.038 | 0.011 | 0.524~0.672 |
| Combined model | 0.500 | 0.039 | 1.000 | 0.424~0.576 |
| **Female 18~45 years** |  |  |  |  |
| Testosterone (ng/dl) | 0.532 | 0.013 | 0.012 | 0.507~0.573 |
| Estradiol (pg/mL) | 0.595 | 0.012 | <0.001 | 0.571~0.544 |
| Progesterone (ng/mL) | 0.551 | 0.012 | <0.001 | 0.527~0.54 |
| Cortisol (μg/dL) | 0.542 | 0.013 | 0.001 | 0.518~0.542 |
| ACTH (pg/mL) | 0.515 | 0.013 | 0.232 | 0.482~0.540 |
| CRP(mg/dL) | 0.593 | 0.013 | <0.001 | 0.569~0.588 |
| Combined model | 0.625 | 0.012 | <0.001 | 0.602~0.573 |
| **Female ≥ 45 years** |  |  |  |  |
| Testosterone (ng/dl) | 0.515 | 0.016 | 0.343 | 0.484~0.545 |
| Estradiol (pg/mL) | 0.535 | 0.015 | 0.023 | 0.505~0.565 |
| Progesterone (ng/mL) | 0.534 | 0.015 | 0.028 | 0.504~0.564 |
| Cortisol (μg/dL) | 0.528 | 0.015 | <0.001 | 0.397~0.457 |
| ACTH (pg/mL) | 0.615 | 0.015 | <0.001 | 0.585~0.644 |
| CRP(mg/dL) | 0.579 | 0.015 | <0.001 | 0.549~0.609 |
| Combined model | 0.617 | 0.015 | <0.001 | 0.588~0.646 |

**Supplemental Table 6. Normal-range values of each indicator in the study.**

| Indicator | Units | Female | Male |
| --- | --- | --- | --- |
| Testosterone | ng/dl | 8.38-35.01 | 197.4-669.6 |
| Estradiol | pg/mL | follicular phase:19.5-144.2  ovulatory period: 63.9-356.7  luteal phase: 55.8-214.2  menopause: ND-32.2 | 0-39.8 |
| Progesterone | ng/mL | follicular phase: ND-1.4  luteal phase: 3.34-25.56  mid-luteal phase: 4.44-28.03  menopause: ND-0.37 | 0.28-1.22 |
| Cortisol | μg/dL | 0-46 | |
| ACTH | pg/ml | AM:5.27-22.45; PM:3.44-16.76 | |
| CRP | mg/dL | 0.00-0.80 | |
